# *Malassezia*-Associated Skin Diseases, the Use of Diagnostics and Treatment

**DOI:** 10.3389/fcimb.2020.00112

**Published:** 2020-03-20

**Authors:** Ditte M. L. Saunte, George Gaitanis, Roderick James Hay

**Affiliations:** ^1^Department of Dermatology, Zealand University Hospital, Roskilde, Denmark; ^2^Department of Clinical Medicine, Health Sciences Faculty, University of Copenhagen, Copenhagen, Denmark; ^3^Department of Skin and Venereal Diseases, Faculty of Medicine, School of Health Sciences, University of Ioannina, Ioannina, Greece; ^4^DELC Clinic, Biel/Bienne, Switzerland; ^5^St. Johns Institute of Dermatology, Kings College London, London, United Kingdom

**Keywords:** *Malassezia*, folliculitis, head and neck dermatitis, seborrheic dermatitis, pityriasis versicolor

## Abstract

Yeasts of the genus, *Malassezia*, formerly known as *Pityrosporum*, are lipophilic yeasts, which are a part of the normal skin flora (microbiome). *Malassezia* colonize the human skin after birth and must therefore, as commensals, be normally tolerated by the human immune system. The *Malassezia* yeasts also have a pathogenic potential where they can, under appropriate conditions, invade the stratum corneum and interact with the host immune system, both directly but also through chemical mediators. The species distribution on the skin and the pathogenetic potential of the yeast varies between different *Malassezia* related diseases such as head and neck dermatitis, seborrheic dermatitis, pityriasis versicolor, and *Malassezia* folliculitis. The diagnostic methods used to confirm the presence of *Malassezia* yeasts include direct microcopy, culture based methods (often a combination of morphological features of the isolate combined with biochemical test), molecular based methods such as Polymerase Chain Reaction techniques, and Matrix Assisted Laser Desorption/Ionization—Time Of Flight mass spectrometry and the chemical imprint method Raman spectroscopy. Skin diseases caused by *Malassezia* are usually treated with antifungal therapy and if there are associated inflammatory skin mechanisms this is often supplemented by anti-inflammatory therapy. The aim of this paper is to provide an overview of *Malassezia* related skin disease, diagnostic methods and treatment options.

## Introduction

Yeasts of the genus, *Malassezia*, formerly known as *Pityrosporum*, are lipophilic yeasts, which are a part of the normal skin flora (microbiome). The genus *Malassezia* belongs to the phylum *Basidiomycota* (class *Malasseziomycetes*) and the genus consists at present of 17 species (Grice and Dawson, 2017; Theelen et al., 2018). It is the most prevalent fungal genus of the healthy skin, but these yeasts also demonstrate a pathogenic potential where they can, under appropriate conditions, invade the stratum corneum. They interact with almost all the cellular constituents of normal epidermis, including keratinocytes, Langerhans cells, melanocytes as well as the host immune system, both directly but also through chemical mediators (Glatz et al., 2015; Grice and Dawson, 2017). *Malassezia* colonize the human skin after birth and must therefore, as a commensal, be normally tolerated by the human immune system. Depending on sampling technique and diagnostic methods they have been isolated from 30 to 100% of newborns (Ayhan et al., 2007; Nagata et al., 2012).

*Malassezia* species are dependent on exogenous lipids because they lack fatty acid synthase genes, except *M. pachydermatis* (Glatz et al., 2015). This explains their distribution on seborrheic skin areas (face, scalp and thorax), but they have been detected from most body sites except the feet (Grice and Dawson, 2017). There is also a correlation between species diversity and anatomical sampling site (Grice and Dawson, 2017; Theelen et al., 2018).

The species distribution on the skin varies between different *Malassezia* related diseases, but their worldwide distribution may also differ (Grice and Dawson, 2017). For example, *M. sympodialis* considered the most prevalent species in Europe and *M. restricta* and *M. globosa* the most predominant species in Asia. The difference in the species distribution may not only be revealed by differences in geographic specificity but may also be due to a difference in diagnostic methods used. Most of the European studies used culture-based methods whereas Asian countries generally have applied molecular based methods and as some *Malassezia* species are slow-growing and more fastidious in culture, such as *M. restricta*, this particular species in culture may be overgrown by a more rapid-growing *Malassezia* species as e.g., *M. sympodialis* (Kohsaka et al., 2018).

Skin diseases caused by *Malassezia* are usually treated with antifungal therapy and if there are associated inflammatory skin mechanisms this is often supplemented by anti-inflammatory therapy. Different *Malassezia* species have shown various antifungal susceptibility patterns (Prohic et al., 2016; Theelen et al., 2018). It may therefore occasionally be important to identify the *Malassezia* species in order to choose the most sensitive antifungal drug although this poses immense practical problems in resource poor settings.

The aim of this paper is to provide an overview of the *Malassezia* related skin diseases Head and neck dermatitis, seborrheic dermatitis, pityriasis versicolor, and *Malassezia* folliculitis, their diagnostic methods and treatment options.

## Diagnostics

Different sampling methods have been used to confirm the presence of *Malassezia* yeasts in skin conditions and these include tape stripping, skin scraping, swabs, and contact plates (Darabi et al., 2009). Direct microcopy is used frequently in clinical settings (Saunte et al., 2018) as it can be used to detect fungal elements after application of potassium hydroxide and adding a dye such as e.g., Parker ink, methylene blue, lactophenol blue, May-Grunwald-Giemsa, Gram staining or a fluorescence dye such as Calcofluor white and Blancophor (Rubenstein and Malerich, 2014; Tu et al., 2018). *Malassezia* is recognized by the detection of characteristic unipolar budding yeasts and in the case of pityriasis versicolor these are accompanied by short hyphae (the so-called spaghetti and meatballs appearance). Hyphae are not detected in head and neck dermatitis and rarely seen in *Malassezia* folliculitis or seborrheic dermatitis/dandruff. Even though it is possible to see differences in the shape of the *Malassezia* yeasts cells as e.g., the globose cells of *M. globosa* or the sympodial budding of *M. sympodialis*, accurate species identification is not possible by direct microscopy. For this, different *in vitro* methods have been applied.

The initial isolation usually employs Dixon's or Leeming-Notman agar and growth at 32–35°C under aerobic conditions. Daily evaluation of the cultures is required to observe the presence of mixed species colonies, which are needed to be separated using needle sampling of the colonies and/or multiple dilutions before subculturing. Identification to species level is achieved by evaluation of the different lipid assimilation profile of the *Malassezia* species (Guého et al., 1996; Mayser et al., 1997) in combination with microscopic morphological features. However, the variations revealed by this conventional mycology approach are not sufficiently specific for the identification of the current expanded *Malassezia* species, as there is a common lipid profile overlap between species (Cafarchia et al., 2011; Theelen et al., 2018). Although these culture-based methods are time-consuming and it is difficult to separate closely related species characteristics of each strain.

For this reason during the last five decades molecular based methods (Arendrup et al., 2013) as well as methods that identify the chemical imprint of the different species e.g., different Polymerase Chain Reaction (PCR) techniques, Matrix Assisted Laser Desorption/Ionization—Time Of Flight (MALDI-TOF) mass spectrometry (Kolecka et al., 2014; Diongue et al., 2018; Honnavar et al., 2018; Saunte et al., 2018) and or Raman spectroscopy (Petrokilidou et al., 2019) have been applied to achieve fast and accurate fungal identification.

Discrepancies in the epidemiological data generated by culture and molecular based *Malassezia* identification methods are well-known and probably reflect differences in growth rate, where the fast growing species may overgrowth slower ones in culture based methods and because molecular based methods are considered to be more accurate (Soares et al., 2015; Prohic et al., 2016). Additionally, species identification using molecular based methods is dependent on reliable “databases” for sequence comparison.

Antifungal susceptibility of *Malassezia* species using agar and broth dilution methods (Clinical & Laboratory Standards Institute and European Committee of Antimicrobial Susceptibility Testing assays) with lipid supplementation has been studied (Cafarchia et al., 2012; Leong et al., 2017; Peano et al., 2017; Rojas et al., 2017). *In vitro* antifungal resistance have been demonstrated in different strains, but as there is no reference procedure for antifungal susceptibility testing the strains may appear susceptible under other test conditions (Peano et al., 2017; Rojas et al., 2017).

Despite the current knowledge of *Malassezia* species' association and contribution to skin disorders, the mechanisms underlying their change from a commensal to pathogen are still to be further elucidated. Furthermore, there is a need for standardization of species diagnostic methods and antifungal susceptibility testing.

## *Malassezia*-Associated Skin Diseases

Even though *Malassezia* is a part of the human microbiome it is also involved in the pathogenesis of head and neck dermatitis, seborrheic dermatitis, pityriasis versicolor, and *Malassezia* folliculitis. It interacts with both the innate and acquired skin immune systems and thereby causes immune reactions under certain conditions. It is possible to detect IgG and IgM antibodies against *Malassezia* in most individuals, but healthy persons are usually not sensitized as is the cases with atopic dermatitis patients. The sensitization can in atopic dermatitis (AD) patients cause a type I hypersensitivity reaction contributing to redness, itching and further scaling in the seborrheic areas of the head and neck, the so-called head and neck dermatitis (Glatz et al., 2015; Kohsaka et al., 2018). In seborrheic dermatitis (Faergemann et al., 2001) the inflammatory reaction that leads to the development of seborrheic dermatitis seems to be an irritant non-immunogenic stimulation of the immune system that leads to complement activation and local increase in NK1+ and CD16+ cells. Pityriasis versicolor is an infection which involves proliferation of the organisms and activation of the formation of hyphae to cause superficial invasion of the stratum corneum.

In *Malassezia* folliculitis the yeasts invade the pilo-sebaceous unit leading to a dilatation of the follicles with large number of *Malassezia* cells. If the follicular walls rupture this results in a mixed inflammatory infiltrate and clinical inflammation.

## Head and Neck Dermatitis

### Epidemiology and Pathogenesis

Head and neck dermatitis is a subtype and difficult to treat form of atopic dermatitis, which is generally seen in post-pubertal atopic dermatitis patients. The prevalence of atopic dermatitis among adults in industrialized countries is 1–3% and it affects 10–20% of children (Brodská et al., 2014). It is thought to be due to a type I hypersensitivity reaction to *Malassezia* antigens (Table 1). The antigens e.g., *M. globosa* protein (MGL_1304) and its homologs from *M. sympodialis* (Mala s 8) and *M. restricta* (Mala r 8) have all been implicated in the pathogenesis of head and neck dermatitis and show different histamine releasing activity (Kohsaka et al., 2018). The *Malassezia* (antigen) proteins are found in sweat and the disease is therefore triggered by sweating (sometimes referred to as sweat allergy) (Hiragun et al., 2013; Maarouf et al., 2018). IgE antibodies against *Malassezia* is found in up to 27% of children and 65% of adults with atopic dermatitis (Glatz et al., 2015).

**Table 1:** *Malassezia* associated diseases and their possible pathogenesis, main diagnostics and differential diagnosis.

**Disease**	**Possible pathogenesis**	**Main diagnostic**	**Examples of differential diagnosis**
Head & neck dermatitis	Type-I hypersensitivity to *Malassezia*	Clinical Skin prick test *Malassezia* spp. specific IgE (Atopy patch test)	Contact dermatitis Steroid induced dermatitis
Seborrheic dermatitis	Colonization with *Malassezia* that triggers irritant dermatitis	Clinical Biopsy shows psoriasiform, spongiotic dermatitis without intraepidermal pustules	Rosacea Sebopsoriasis Systemic lupus erythematois Tinea capitis Zinc deficiency Contact dermatitis
Pityriasis versicolor	*Malassezia* infection	Clinical Direct microscopy with unipolar budding yeast and hyphae (spaghetti and meatballs)	Vitiligo Pityriasis alba Chloasma Nummular dermatitis
*Malassezia* folliculitis	Invasion of the pilo-sebaceous with *Malassezia*	Histopathology Direct microscopy with unipolar budding yeast (rarely hyphae)	Acne Steroid acne Bacterial folliculitis Eosinophilic folliculitis Pustular drug eruptions Lymphomatoid papulosis

*Malassezia's* interaction with the skin immune system is thought to be both humoral and cell-mediated and it contributes to and accentuates the pre-existing skin inflammation in AD (Brodská et al., 2014). It is suggested that an increased pH, which is higher in AD patients, may contribute to allergen release by *Malassezia*. The disturbed skin barrier in AD allows both *Malassezia* allergens as well as cells to penetrate the epidermis and hereby introducing them to toll-like receptor 2 on dendritic cells and keratinocytes. A release of pro-inflammatory cytokines and *Malassezia* spp.- specific IgE antibodies is produced through T cell mediated activation of B cells and through dendritic cells and mast cells and this contributes to the skin inflammation. Furthermore, autoreactive T cells may cross react and sustain skin inflammation (Glatz et al., 2015).

### Clinical Presentation

**Figure 1:**
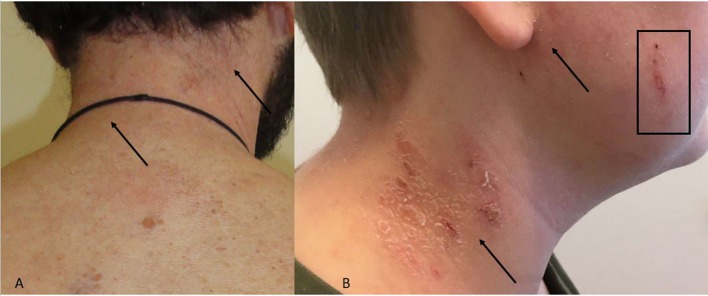
**(A,B)** Head and neck dermatitis. **(A)** Neck with erythema and discrete skin scales. Arrows indicate the area. **(B)** Skin scales, erythema (arrows) and excoriation (square) of neck and cheek.

The clinical manifestations of head and neck dermatitis are typically erythematous involvement of the eyelids, forehead and neck; sometimes the changes are wheal-like (urticarial) (Maarouf et al., 2018). Affected areas are itchy and there is often scaling giving the appearances of an eczema flare (Figures 1A,B).

### Diagnosis

The diagnosis is based upon the clinical picture and may be supported a positive type I allergic reaction to *Malassezia* and a positive skin prick test with *Malassezia* spp. –specific extract is found in 30–80% of adult atopic dermatitis (Glatz et al., 2015). A study by Devos and van der Valk found that all AD patients with head and neck dermatitis had increased *Malassezia-spp*. specific IgE as compared with only 13.6% of AD patients without head and neck dermatitis (Devos and van der Valk, 2000). A commercial and standardized kit (ImmunoCAP® m70, Phadia) is available for measuring *Malassezia spp*.-specific serum IgE (Glatz et al., 2015). The use of atopy patch test shows diverse results (Brodská et al., 2014). In two different studies (Ramirez De Knott et al., 2006; Johansson et al., 2009) there was no correlation between IgE and atopy patch test for *Malassezia*, whereas Johansson et al. (Johansson et al., 2003) found that atopic patch test was positive in 30% of AD patients without head and neck dermatitis and in 41% of patients with head and neck dermatitis.

### Treatment

Head and neck dermatitis can be treated using anti-inflammatory medications, antifungals or a combination.

The main purpose of the antifungal treatment is to reduce the skin colonization thereby reducing the amount of allergen causing the type I hypersensivity. It has been shown that AD patients with head and neck dermatitis treated with anti-fungals (itraconazole) show decreases in the total *Malassezia* specific IgE, eosinophil count as well as improving clinical severity scores (Ikezawa et al., 2004).

The clinical improvement is usually seen within the first week(s) and the daily regimen is often continued for 1–2 months followed by a twice weekly regimen to prevent relapse (Darabi et al., 2009). Systemic antifungals are useful in severe cases or when treatment failure after topical therapy.

Furthermore, in AD patients repair of the impaired skin barrier and a reduction of the inflammation with e.g., calcineurin inhibitors or topical steroids are very useful (Nowicka and Nawrot, 2019). It is not clear if the reduction of the inflammation is more important than reducing skin colonization of *Malassezia* for two reasons. First of all the treatment responses to hydrocortisone combined with placebo shampoo compared with miconazole-hydrocortisone cream and ketoconazole shampoo are not significantly different (Broberg and Faergemann, 1995). Secondly, some antifungals have anti-inflammatory properties (inhibit IL-4 and IL-5 production) (Kanda et al., 2001).

## Seborrheic Dermatitis

### Epidemiology and Pathogenesis

Seborrheic dermatitis is an inflammatory dermatosis with a predilection for anatomical areas with high sebaceous gland concentration such as the midface, chest, back, and scalp. Seborrheic dermatitis located on the scalp and dandruff should be considered as representing different ends of a disease severity spectrum (Grimalt, 2007). Therefore, for scalp disease the term seborrheic dermatitis/dandruff complex is suggested to encompass the scaling both with inflammation (seborrheic dermatitis) and without inflammatory component (dandruff). As dandruff is extremely common and practically all adults are affected at some point in their life, we will note only relevant data in the pathogenesis section that help us to understand seborrheic dermatitis.

Seborrheic dermatitis is a relative common dermatosis and few recent meticulous studies have addressed the point prevalence of this disease. Thus the point prevalence of seborrheic dermatitis in 161,269 working individuals in Germany (Zander et al., 2019) was recorded to be 3.2% with seborrheic dermatitis being three times more common in men than in women. Also, seborrheic dermatitis prevalence increased with age (2.0% in <35 years; 3.6% in 35–64 years; 4.4% ≥65 years) and there was an association with other fungal diseases such as tinea pedis, onychomycosis and pityriasis versicolor. The age dependence of seborrheic dermatitis is probably responsible for the increased prevalence (14.3%) recorded in the Rotterdam study (Sanders et al., 2018a) as the median age of patients was 67.9 years. These robustly acquired data confirm the association of seborrheic dermatitis with gender (two-fold increase in men), season (increased in winter) and generalized xerosis cutis. A darker skin phenotype was a protective factor for seborrheic dermatitis. Whether this was due to difficulty in recording erythema in darker skin types or the fact that it represents a different barrier function in these skin phenotypes is a matter of debate. Nevertheless seborrheic dermatitis was also commonly diagnosed in 2.1% of young Korean male army recruits (Bae et al., 2012) (93.3% of cohort between 19 and 24 years of age), supporting the generally suggested prevalence of seborrheic dermatitis between 2 and 8% (Palamaras et al., 2012).

It well established that seborrheic dermatitis prevalence is significantly increased in subgroups of patients such as those with Human Immunodeficiency Virus (HIV) infection, where it is associated with low CD4 counts (Lifson et al., 1991) as well as neurological patients. These include those with Parkinson's disease (Skorvanek and Bhatia, 2017) patients as well as patients with spinal cord injury on which seborrheic dermatitis appears above the level of injury (Han et al., 2015), pointing toward brain-skin axis involvement. In the light of the recent implication of *Malassezia* yeasts in pancreatic ductal carcinoma development (Aykut et al., 2019), these epidemiological observations point to future research areas (Laurence et al., 2019). The understanding of the pathogenesis of seborrheic dermatitis is limited by the overlap with other conditions such as psoriasis (sebopsoriasis), the indistinct borders between seborrheic dermatitis and dandruff and the absence of a robust severity scoring system. Thus, findings in dandruff pathophysiological changes that are generated from scalp are not necessarily applicable to facial seborrheic dermatitis. Likewise only recently markers to differentiate the overlapping cases of psoriasis and seborrheic dermatitis (sebopsoriasis) have been developed. These include immunohistochemistry markers that address clinical and pathological indistinct cases of sebopsoriasis (Cohen et al., 2019). Additionally, seborrheic dermatitis patients do not share susceptibility loci with psoriasis patients (Sanders et al., 2018b). Regarding the implication of *Malassezia* yeasts in the pathogenesis of seborrheic dermatitis and dandruff there are characteristic and persistent findings that link seborrheic dermatitis or dandruff associated *Malassezia* strains with the respective conditions. Thus *M. furfur* strains isolated from seborrheic dermatitis lesions produce, *in vitro*, significantly more bioactive indolic substances as compared to strains isolated from healthy skin (Gaitanis et al., 2008). These substances [i.e., indirubin, 6-formylindolo[3,2-b]carbazole (FICZ), indolo[3,2-b]carbazole (ICZ), malassezin, and pityriacitrin] are also found on seborrheic dermatitis skin and correspond to the most active aryl-hydrocarbon receptor ligands known (Magiatis et al., 2013). As a marker of their clinical significance, indirubin is used as a potent local treatment for psoriasis (Lin et al., 2018), while there are ongoing clinical trials that evaluate aryl hydrocarbon receptor ligands applied locally for this disease (https://clinicaltrials.gov/ct2/show/NCT04053387). Likewise, the irritating effect on the skin through a compromised permeability barrier function (Turner et al., 2012) of free fatty acids (DeAngelis et al., 2005) and squalene peroxides (Jourdain et al., 2016) produced by *Malassezia* lipases as a result of its nutritional needs, are key players, at least, in the pathogenesis of dandruff. Accordingly, the skepticism expressed (Wikramanayake et al., 2019) on the implication of *Malassezia* yeasts in seborrheic dermatitis can be a useful starting point for future research toward the better understanding of seborrheic dermatitis pathogenesis.

### Clinical Presentation

**Figure 2:**
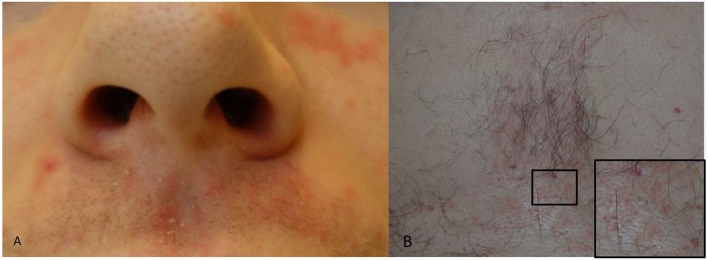
**(A,B)** Seborrheic dermatitis. **(A)** Peri-nasal skin and upper lip with erythema and greasy skin scales. **(B)** Erythema and greasy skin scales of the chest and a close-up (square) of an area with erythematous lesions.

Seborrheic dermatitis presents with erythema, small papules and sometime pustules overlayed with greasy, white to yellow scales. The areas of predilection include the nasolabial folds and the upper lip close to the nostrils (Figure 2A), the eyebrows and the root of the nose, the pre- and retro auricular areas, the sternum (Figure 2B) and less often the back. Scalp seborrheic dermatitis/Dandruff does not involve the whole scalp, rather it appears as patchy areas of erythema and scaling. Involvement of the eye presents as seborrheic dermatitis blepharitis.

### Diagnosis

The diagnosis of seborrheic dermatitis is mostly clinical. The typical cases are straightforward in their recognition while some confusion can be created when there is co-existence with rosacea or late-onset acne. In rosacea the involvement of “convex” anatomical areas (nose, cheeks) and the evaluation of precipitating factors is of help. In acne the lesions are located in the hair follicles, scaling unless receiving therapy is not prominent and the prevailing lesions are comedones, papules and pustules.

Biopsy should be restricted to difficult to diagnose cases and the appearances are mostly described as a psoriasiform, spongiotic dermatitis without intraepidermal pustules (Table 1). Routine cultures for identification and characterization of *Malassezia* species involved to a case of seborrheic dermatitis are not currently suggested. Hopefully in the future, our understanding of seborrheic dermatitis pathogenesis could be associated with identification of virulence factors of *Malassezia* yeasts. This could possibly lead to the development of therapy guided by the pathogenetic mechanisms (tryptophan metabolism, enzyme production) of the case related *Malassezia* strain.

### Treatment

The patient should be informed that seborrheic dermatitis can be a chronic, recurring condition and side-effects of long-term treatment should be weighed against the potential gain. This mostly pertains to topical steroids that are used in clinical practice to rapidly reduce erythema (Gupta and Versteeg, 2017). When long-term control of the inflammatory response in seborrheic dermatitis is required topical use of the calcineurin inhibitors tacrolimus and pimecrolimus is advised (Ang-Tiu et al., 2012). Safety regarding carcinogenicity of these substances is extrapolated from data in atopic dermatitis and does not seem a reason of concern (Cook and Warshaw, 2009). The use of topical antifungals (ketoconazole, ciclopirox) is supported by recent systematic reviews (Okokon et al., 2015) and given their high efficacy and improved safety they should be included in relevant therapeutic schemes. Also it should be stressed that both pimecrolimus and tacrolimus have antifungal action against *Malassezia* yeasts (Sugita et al., 2006) so at least part of their activity in seborrheic dermatitis can be attributed to this. A variety of alternative or natural product treatments are also suggested for seborrheic dermatitis (Gupta and Versteeg, 2017) while a recent suggestion is the use of formulations that restore the barrier function of the skin (Purnamawati et al., 2017) and definitely formulations that restore the barrier function of the skin will be a useful addition to treatment (Wikramanayake et al., 2019). Furthermore various salts are also efficient, like lithium succinate, which seems to interfere with the availability of the prerequisite lipids for *Malassezia* growth (Mayser and Schulz, 2016). Systemic antifungals are suggested for resistant or rapidly relapsing cases of seborrheic dermatitis (Gupta et al., 2014).

## Pityriasis Versicolor

### Epidemiology and Pathogenesis

Pityriasis versicolor is a mild, chronic infection of the skin caused by *Malassezia* yeasts, characterized by discrete or confluent, scaly, dark or depigmented patches, mainly on the upper trunk but this can extend to the neck, abdomen and other sites, although the peripheries are usually spared.

Pityriasis versicolor occurs in both tropical, where it may be very common, and temperate climates and affects both genders equally. However, lesions in temperate areas are often noticed after a visit to a warmer environment. It is commonest in teen-agers and young adults but can occur at any age. Data on global prevalence is not available, however in tropical climates, the condition is more common than in temperate zones, and in one study from Bahia, Brazil 40% of the population of some areas was affected (Santana et al., 2013). Although there are reports of an association between pityriasis versicolor and a number of other underlying conditions, it generally occurs in otherwise healthy individual although patients with idiopathic and iatrogenic Cushing's syndrome are more susceptible (Finding et al., 1981). It does not appear to be more common in the acquired immune deficiency syndrome (AIDS) (Mathes and Douglass, 1985).

A striking feature of most cases of pityriasis versicolor is the presence of hyphae in lesions. But the reasons for hyphal growth are still unknown. The activation of the MGL_3741 gene which encodes the enzyme Dihydroxy acid dehydratase (DHAD) in *M. globosa* has been implicated as it is present in lesional but not non-lesional skin (Aghaei Gharehbolagh et al., 2018) Lack of inflammation in lesions of pityriasis versicolor is noticeable although there is evidence of interaction between *Malassezia* species in this condition and innate and acquired immunity (Brasch et al., 2014) T-cell inhibition by a lipid component associated with the yeast cell wall has also been reported (Kesavan et al., 1998) which may partially explain the lack of clinically significant inflammation.

**Figure 3:**
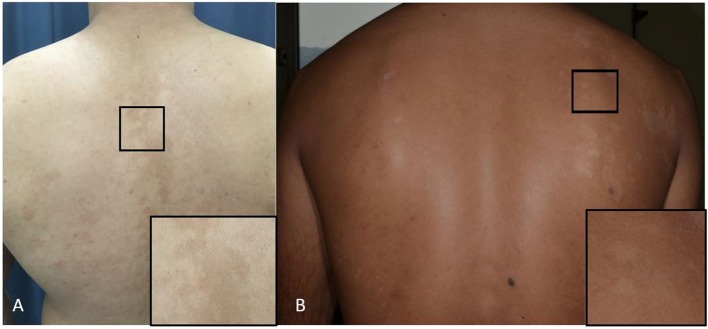
**(A,B)** Pityriasis versicolor. **(A)** Hyperpigmented maculae on the back and a close-up of the lesion (square). **(B)** Hypopigmented maculae and a close-up of the lesion (square).

The mechanism for the typical pigmentary changes seen in pityriasis versicolor is still not understood, although electron microscopy shows abnormally large melanosomes in hyperpigmented lesions (Figure 3A), and smaller-than-normal melanosomes in hypopigmented ones (Figure 3B). Depigmentation has been explained on the production of dicarboxylic acids produced by *Malassezia* species (e.g., azaleic acid) causing competitive inhibition of tyrosinase and perhaps a direct cytotoxic effect on hyperactive melanocytes (Nazzaro-Porro and Passi, 1978). *M. furfur* produces pigments and fluorochromes with tryptophan as sole nitrogen source. They (i.e., malassezin, pityriacitrin, pityrialacton, pityriarubins) may explain some clinical phenomena of pityriasis versicolor (depigmentation. fluorescence, lack of sunburn in pityriasis versicolor alba) (de Hoog et al., 2017).

The *Malassezia* species mainly identified in pityriasis versicolor lesions are *M. globosa* and also *M. sympodialis* and *M. furfur*.

### Clinical Presentation

The primary lesions are well demarcated macules, which may be slightly erythematous and covered by fine scales which may only be noticeable after scratching the lesional surface. These co-alesce to form scattered patches of hypo- or hyperpigmentation (Figures 3A,B). Itching is very mild. The sites most commonly affected are the upper trunk, but there is often spread to the upper arms, the neck and the abdomen. Lesions in the axillae and groins, and on the thighs and genitalia occur, and extension down the forearms on to the backs of the hands; these atypical forms of pityriasis versicolor may be associated with oval yeast forms seen in direct microscopy. Another rare but well documented variant is one where there is marked atrophy or anetoderma-like change in the skin that follow infection (Tellechea et al., 2012). Pityriasis versicolor is a chronic infection if left untreated. In some patients, lesions recur rapidly and may not respond well to treatment. Such cases, while not common, are seen regularly. Some have been associated with the presence of the organism, *M. japonica*, and raised IgE levels (Romero-Sandoval et al., 2017).

Vitiligo and chloasma are normally distinguishable from pityriasis versicolor by their complete absence of scaling.

### Diagnosis

Under filtered ultraviolet (Wood's) light, the scaly lesions may show pale yellow fluorescence. Direct microscopy shows coarse mycelium, fragmented into short filaments, together with spherical, thick-walled yeasts. Occasionally, only oval yeasts may be seen (see above). The characteristic appearance on microscopy has been described as “spaghetti and meatballs” (Table 1). Detection of *Malassezia* species by culture or molecular methods from skin scrapings is of no diagnostic value, and does not form part of the diagnostic investigation of pityriasis versicolor. Dermoscopy, although useful in confirming the scaling, does not identify specific diagnostic features (Mathur et al., 2019).

### Treatment

The first line treatment is topical antifungal therapy. The topical azole antifungals work well in pityriasis versicolor, and there is no significant difference in results achieved by different azoles. The usual time to recovery is 2–3 weeks. A practical problem with the use of topical antifungals is the difficulty of applying creams to a wide body surface area. An alternative solution to this is ketoconazole shampoo which is lathered into the skin in a shower and then washed off after 3–4 min, and although it has not been fully evaluated in pityriasis versicolor, two or three applications of the shampoo appear to clear most infections. Terbinafine 1% cream, but not oral terbinafine, is also effective. Another approach is the application of 2.5% selenium sulfide in a detergent base (Selsun® shampoo). It is applied to all the affected areas and left overnight. Alternatives include 50: 50 propylene glycol in water. The latter has also been used intermittently as long-term suppressive therapy to prevent relapse (Faergemann and Fredriksson, 1980).

Oral itraconazole is also very effective in cases of pityriasis versicolor 100 mg daily for 10 days (Delescluse, 1990) although it is usually given in extensive or recalcitrant cases. Fluconazole has also been used.

Whatever medication is given patients should be warned that normalization of pigmentation may take several months after the end of treatment.

## *Malassezia* Folliculitis

### Epidemiology and Pathogenesis

*Malassezia* folliculitis is an inflammatory condition caused by *Malassezia* yeasts involving the pilo-sebaceous unit.

Predisposing factors includes immunosuppression (e.g., immunosuppressive medication, broad spectrum antibiotics, diabetes, HIV, hematological malignancies), occlusion and sweating (Tragiannidis et al., 2010; Prohic et al., 2016). It is more frequent in, or after visiting, tropical areas or hotter climates because of humidity and high temperatures (Tragiannidis et al., 2010).

The most prevalent species associated with *Malassezia* folliculitis are *M. globosa, M. restricta* and *M. sympodialis* (Akaza et al., 2009; Ko et al., 2011; Durdu et al., 2013; Prohic et al., 2016).

### Clinical Presentation

**Figure 4:**
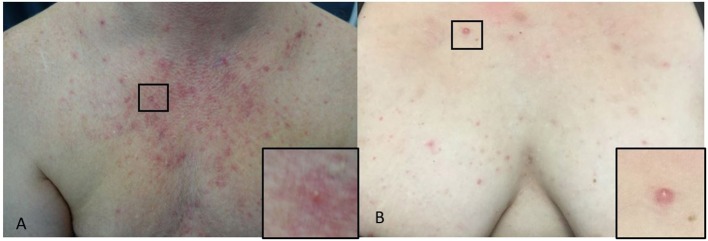
**(A,B)** *Malassezia* folliculitis. **(A)** Erythematous paplues and pustules on the chest of a male and a close-up of a papule (square). **(B)** On the chest of a woman and a close-up of a papule (square).

The typical presentation is monomorphic, approximately 2–4 mm, erythematous itchy papules or papulopustules on the chest (Figures 4A,B), back, upper arms, neck and face; some patients have concomitant pityriasis versicolor or seborrheic dermatitis (Hald et al., 2014). *Malassezia* folliculitis, especially in adolescent, may be misdiagnosed as acne or bacterial folliculitis, but comedones are absent and itching is a common symptom (Hald et al., 2014; Tsai et al., 2019). The itching may be less pronounced in immunosuppressed patients (Hald et al., 2014).

### Diagnosis

The diagnosis is based upon the clinical picture and symptoms supported by mycological detection and response to antifungal therapy (Prohic et al., 2016). Histopathology can be used to differentiate *Malassezia* folliculitis from other types of folliculitis such as e.g., bacterial, eosinophilic or pustular drug eruptions. In *Malassezia* folliculitis invasion and dilatation of follicles with large number of *Malassezia* conidia (and rarely hyphae) is seen and inside the follicle there is a reticular pattern of keratin plugging in the majority of patients (An et al., 2019). The follicular walls may rupture resulting in a mixed inflammatory infiltrate of neutrophils, lymphocytes and histiocytes in the dermis. Direct microscopy on skin scraping and the content of pustules treated with KOH (and a dye) will detect unipolar budding yeast, rarely hyphae (Table 1). In a study by Tu et al. Gram staining has been shown to have a sensitivity and specificity of 84.6 and 100% as compared with a final diagnosis of *Malassezia* folliculitis when two of three criteria was met: 1. Typical clinical presentation, 2. Biopsy with *Malassezia* in inflamed hair follicle, 3. Treatment response to antifungal therapy (Tu et al., 2018). This suggests that direct microscopy which is both rapid, simple and non-invasive is an alternative to histology. Nevertheless, direct microscopy is not species specific as are culture- or molecular-based methods and it does not reveal location of the fungus in relation to the follicle.

Other diagnostic methods includes Wood's lamp which fluorescence yellow-green when the lesions is illuminated, reflectance confocal microscopy and optical coherence tomography (Rubenstein and Malerich, 2014; Andersen et al., 2018).

In clinical settings initial diagnosis based upon the combination of symptoms such as itch, clinical picture with monomorphic papulopustules without comedones supported by direct mycological detection by microscopy is sufficient to initiate therapy while awaiting histopathology results. The direct microscopy is important to differentiate *Malassezia* folliculitis from bacterial folliculitis.

### Treatment

Systemic itraconazole 100–200 mg daily has been used for 1–4 weeks with a clinical treatment effect of 69–100% (Parsad et al., 1998; Durdu et al., 2013; Suzuki et al., 2016; Tsai et al., 2019) and fluconazole 100−200 mg daily for 1–4 weeks with a clinical effect of 80% (Rhie et al., 2000). Combination of systemic antifungals and topical antifungals (Abdel-Razek et al., 1995; Prindaville et al., 2018) or tretinoin/bensylperoxide (Ayers et al., 2005) is also useful. Topical therapies which have proven useful for the treatment of *Malassezia* folliculitis include azoles (Back et al., 1985; Rhie et al., 2000; Suzuki et al., 2016; Prindaville et al., 2018; Tsai et al., 2019), selenium sulfide once daily for 3 days then weekly (Back et al., 1985) and propylene glycol 50 % twice daily (Back et al., 1985). Systemic antifungal monotherapy is thought to be more efficient than topical monotherapy, but in a small study (*N* = 44) comparing ketoconazole cream twice daily with oral itraconazole 100 mg daily an improvement and treatment respond was noted in both groups although the topical treatment required a longer treatment course (Suzuki et al., 2016). Topical therapy may therefore be useful and considered in patients as a prevention measure or in patients with contraindication for systemic therapy.

Recurrence is common after treatment is completed, and maintenance therapies such as weekly topical or monthly oral antifungals have been used as prevention measures (Levy et al., 2007; Rubenstein and Malerich, 2014).

Alternative treatment options include photodynamic therapy (Lee et al., 2010, 2011).

Currently, there is no internationally approved treatment guideline for the management of *Malassezia* folliculitis.

## Conclusion

The *Malassezia* yeasts are complex fungi which are part of the normal skin microbiome. They have pathogenic potential and are able to cause skin related diseases through different mechanisms: an activation of the immune system as in head and neck dermatitis, an eczematous/inflammatory reaction as in seborrheic dermatitis, an infection of stratum corneum as in pityriasis versicolor or a colonization (invasion) with a large number of *Malassezia* yeasts of the pilo-sebaceous unit as in *Malassezia* folliculitis. To support the clinical suspicion of the association between *Malassezia* and disease, a broad spectrum of techniques is used for the confirmation of the presence of *Malassezia* yeasts or for the detection of pathogenetic mechanisms such as *Malassezia* related type I allergy. Traditional direct microscopy, culture on lipid enriched media, biochemical tests and histopathology but also newer molecular based methods can be used for the detection of *Malassezia* yeast. For confirmation of type I allergy to *Malassezia* a specific IgE testing or prick testing is useful. A positive treatment response to antifungals, backed by reduction or temporary elimination of the organisms is highly suggestive, if not confirmatory, of a *Malassezia* etiology, but there are other variables such as the host's general condition and the species involved. Further investigative work that helps to delineate the disease mechanisms and the role, if any, of other members of the skin microbiome in the process is needed.

## Ethics Statement

For patients providing clinical photos a written consent was obtained.

## Author Contributions

DS, GG, and RH planned, wrote, and contributed to the critical review of the manuscript.

### Conflict of Interest

DS was paid as a consultant for advisory board meeting by AbbVie, Janssen, Sanofi, and Leo Pharma. Leo Pharma and received speaker's honoraria and/or received grants from the following companies: Abbvie, Galderma, Astellas, Novartis and Leo Pharma during the last 3 years. The remaining authors declare that the research was conducted in the absence of any commercial or financial relationships that could be construed as a potential conflict of interest.

## Abbreviations

AD: atopic dermatitis
*M*: *Malassezia*
HND: Head and neck dermatitis
PCR: Polymerase Chain Reaction
PV: pityriasis versicolor
SD: seborrheic dermatitis.
